# Unveiling Tobacco Vendor Practices: Assessing the Knowledge of and Compliance With Cigarettes and Other Tobacco Products Act (COTPA) Sections Five and Six at the Point of Sale in Raebareli, India

**DOI:** 10.7759/cureus.96533

**Published:** 2025-11-10

**Authors:** Sourabh Paul, Neeraj Pawar, Abhay Singh, Mukesh Shukla, Bhola Nath

**Affiliations:** 1 Department of Community and Family Medicine, All India Institute of Medical Sciences, Raebareli, Raebareli, IND

**Keywords:** compliance, cotpa, point of sale, tobacco control, vendor knowledge, youth access

## Abstract

Background

Tobacco use is one of the major public health issues in India, with 28.6% of adults using tobacco according to the Global Adult Tobacco Survey-2. The Cigarettes and Other Tobacco Products Act (COTPA) 2003 aims to curb tobacco use through restrictions on advertising (section five) and sales to minors and near educational institutions (section six). This study assessed tobacco vendors’ knowledge and compliance with these provisions in Raebareli, Uttar Pradesh, a tier II city, to identify gaps in implementation.

Methods

A community-based cross-sectional study was conducted from July to December 2023 in Raebareli’s 35 municipal wards, covering 2.8 lakh residents. Using convenience sampling, 244 tobacco vendors were included. Data were collected via a structured proforma containing vendor demographics, knowledge of COTPA (12 questions), and compliance with sections five and six (nine questions). Knowledge and compliance scores were categorized as adequate (>50%) or inadequate. Univariate and multivariable logistic regression analysed associations with sociodemographic factors and operational profile of the shops, using IBM SPSS Statistics for Windows, Version 20 (Released 2011; IBM Corp., Armonk, New York, United States).

Results

Of 244 vendors, 197 (80.7%) had inadequate knowledge, with only seven (2.9%) aware of the 100-yard school distance rule and awareness about the single cigarette sale ban was nil. Compliance was inadequate in 120 (49.2%) vendors, with 229 (93.9%) openly displaying tobacco and only four (1.6%) showing signage prohibiting sales to minors. Higher education (adjusted odds ratio or AOR=4.117, p=0.011) and exclusive tobacco vending (AOR=2.606, p=0.014) predicted better knowledge, while permanent shops (AOR=2.343, p=0.036) and mixed-product sales (AOR=2.093, p=0.027) predicted higher compliance.

Conclusion

Low knowledge and compliance with COTPA in Raebareli highlight enforcement gaps. Targeted education and stricter monitoring are needed to enhance adherence and compliance.

## Introduction

Tobacco use continues to pose a major public health challenge both globally and in India, as it contributes to various non-communicable diseases. According to the Global Adult Tobacco Survey (GATS-2, 2016-17) approximately 28.6% of adults in India use some form of tobacco, highlighting the scale of the issue [1]. To address this, the Government of India enacted the Cigarettes and Other Tobacco Products Act (COTPA) 2003, a comprehensive tobacco control law aimed at reducing tobacco use through various regulatory measures [2].

Among the different sections of the COTPA, section five prohibits direct or indirect advertisement, promotion, and sponsorship of tobacco products, aiming to limit exposure and influence, especially among the youth. Section six restricts the sale of tobacco products to individuals below 18 years of age and within 100 yards of educational institutions [2]. These provisions align with the World Health Organization’s Framework Convention on Tobacco Control (FCTC), specifically article 13, which bans tobacco advertising, promotion, and sponsorship, and article 16, which addresses sales to and by minors [3]. COTPA restricts point of sale (POS) tobacco promotion as follows: (1) advertisements are only allowed at the POS; (2) limited to two or fewer tobacco advertisements; (3) the size of advertisement boards can be no more than 60 cm by 90 cm; (4) the content of advertisements is limited to only the brand name and product image; and (5) at least 25% of the surface area of tobacco advertisements must include a health warning [4].

Tobacco POS restrictions aim to curb tobacco use by limiting marketing and sales, but effectiveness depends on compliance, which varies globally [5,6]. It has also been highlighted that there is an inverse relationship between implementation of tobacco control policy compliance and tobacco use [4]. Meta-analysis about the implementation of COTPA Act section five and six a shows various regional variations [7].

It was also demonstrated by the Euro monitor report 2013 that 85% of all tobacco sales of all tobacco products take place through tobacco vendors [8]. Similarly, tobacco use tends to increase with age, but in high-prevalence areas, initiation can start early age. Tobacco vendors’ behaviour significantly influences consumer’s (specially children) access to tobacco products. When vendors question minors or request identification, it deters purchases due to fear of being caught or reported to parents [9].

The success of any law depends on various stakeholders fulfilling their specific roles and responsibilities. Tobacco vendors, as key intermediaries between consumers and manufacturers, are critical stakeholders in this process. To effectively implement the COTPA, the focus should be on raising awareness and fostering a positive attitude among stakeholders toward the legislation. There are very few studies conducted in tier II cities of India to highlight the vendor awareness about COTPA and its association with compliance at point of sale [7-9]. So the present study was conducted to evaluate tobacco vendors' knowledge of COTPA, assess compliance with COTPA sections five and six at POS, and investigate the associations of knowledge and compliance with various sociodemographic variables and the operational profile of shops in Raebareli city, Uttar Pradesh.

## Materials and methods

Study design and setting 

The present study was a community-based cross-sectional study conducted in the municipal area of the district Raebareli of Uttar Pradesh. The municipal area, which spans 4,043 square kilometres and has a total population of 2.8 lakh, is divided into 35 wards, each serving as a territorial constituency [10].

Study duration

The study was conducted between July and December 2023.

Study population

All the vendors selling different types of tobacco products in the study area and who had given written informed consent for participation were included in the study. Big shops and malls selling tobacco were excluded.

Sample size

A study conducted by Venugopal et al. [11] in Chennai, India, found that 18% of the tobacco vendors knew that an open display of tobacco products should not be done. Considering P=18.0%, Confidence interval of 95%, and absolute precision (d) of 5%, the total sample size derived was 236.

The formula used for sample size calculation was: n= z ²₁₋ α/₂P (1-P)/ d², where n=Total sample size, P=Prevalence, d=absolute precision, 1- α/2=Confidence interval.

Sampling design

**Figure 1:**
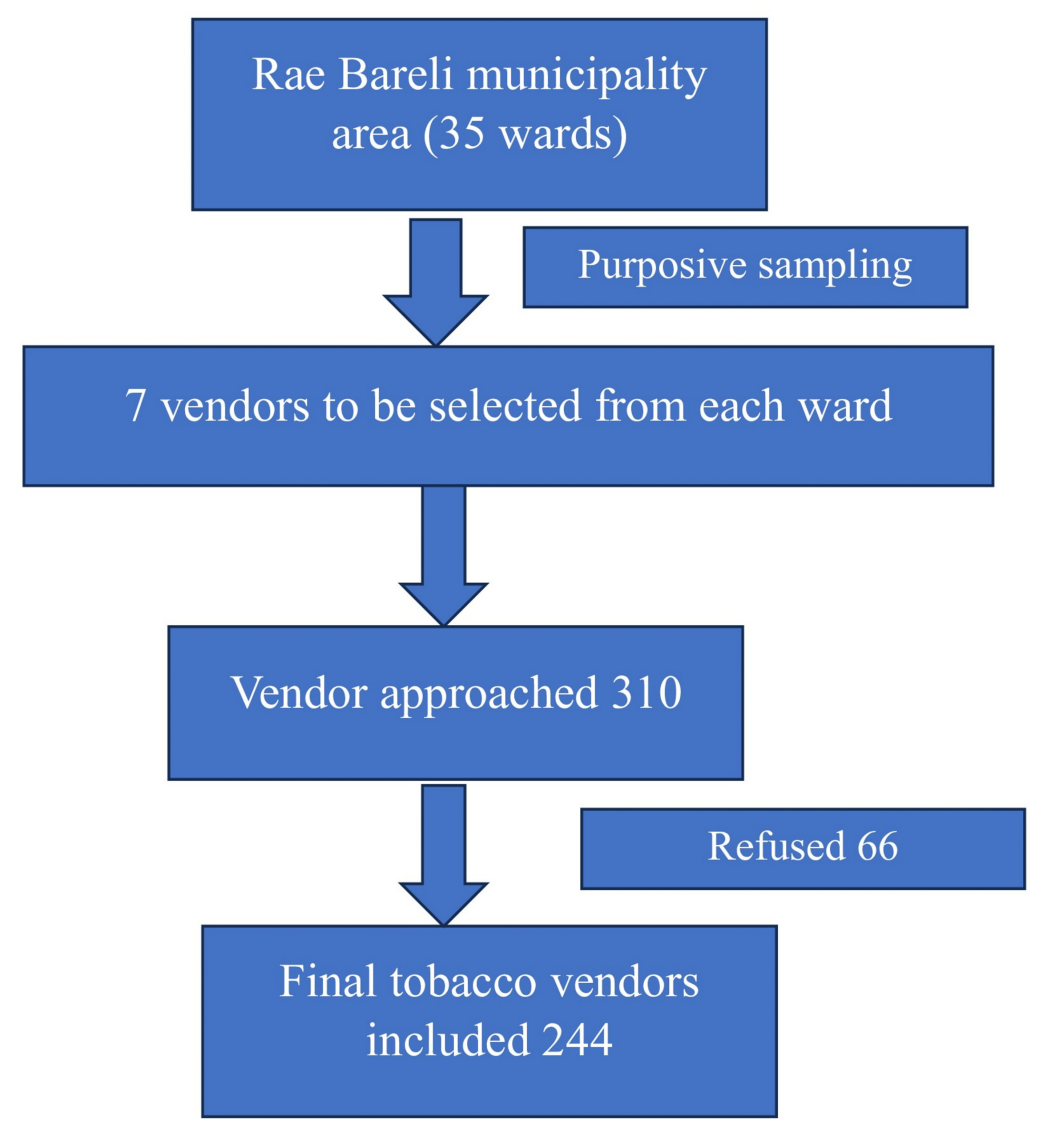
Flow diagram of the sampling method

Raebareli municipal area has 35 wards. Tobacco vendors were selected using convenience sampling from each ward. To reach the required sample size, seven vendors were approached from each ward. However, because of their refusal to participate (21.0%), extra vendors were approached from the same ward. A total of 310 vendors were initially approached, and after considering refusal, a final sample size of 244 was reached (Figure 1).

One road in a specific ward was selected, and in that road a particular tobacco vendor's shop was selected. From there, the investigator went on searching till the required number of shops was achieved.

Study tool

A self-designed and structured proforma was used to collect data from the vendors. The proforma had three sections. The first section contained demographic characteristics of the vendor and operational profile of the shop, the second section contained closed-ended questions pertaining to the knowledge of the tobacco vendors about COTPA (12 questions), and the third section was about compliance assessment (section five and six) and had nine questions for observation. Pilot testing was done among 30 vendors outside the study area for testing face validity, and based on the findings two questions were modified. Content validity of the knowledge questionnaire was checked among five experts and the content validity index (ICVI) was 0.921.

Data collection method

Data collection from the tobacco vendors was done using the structured proforma and the observation schedule. The data collectors were trained before data collection. The vendors were interviewed after consent about their knowledge of the COTPA using the structured proforma and at the same time the data collector assessed the vendor's shop, its surroundings, and his practice, relating it to compliance with the act using an observation checklist. The components of observation checklist were: number of promotional materials, size, health warning sign, selling of tobacco to minors, etc.

Statistical analysis

The data collected was entered in MS Excel (Microsoft Corp., Redmond, WA, US) and analyzed using IBM SPSS Statistics for Windows, Version 20 (Released 2011; IBM Corp., Armonk, New York, United States). The descriptive data was presented as proportions. Each knowledge question had one point for the correct answer, so the maximum attainable score was 12. Similarly, each compliant assessment question to sections five and six of the COTPA had one point, so the maximum attainable points were nine. The total knowledge as well as compliance score was categorized into two groups based on the 50% score. Univariate and multivariable logistic regression was performed to determine factors associated with high knowledge and compliance score. The association was represented as a crude Odds Ratio (OR) and Adjusted Odds Ratio (AOR) with a 95% confidence interval (CI). A P-value of 0.05 was considered for determining the factors associated with high scores in the multivariable logistic regression model.

Ethical issue

The study was conducted after receiving approval from the Bioethics Cell, All India Institute of Medical Sciences, Raebareli (approval 2023-11- IMP-5). Written informed consent from the participants was taken and confidentiality was maintained.

## Results

Table 1 shows the demographic characteristics of the participants and the operational profile of the shops.

**Table 1:** Socio-demographic characteristics of tobacco vendors and operational profile of the shops (n=244) Data has been represented by n and % where n is the number of vendors.

Variable	Number (%)
Age of the vendor (yrs.)	
<35	70 (28.7)
≥35	174 (71.3)
Gender	
Female	41 (16.8)
Male	203 (83.2)
Educational status	
Illiterate	55 (22.5)
Up to primary	34 (13.9)
Up to high school	42 (17.2)
Up to higher secondary	54 (22.2)
Graduate & above	59 (24.2)
Duration of running the shop (yrs.)	
<10	70 (28.7)
≥10	174 (71.3)
Type of shop	
Movable or temporary	33 (13.5)
Permanent or fixed	211 (86.5)
Type of seller	
Only tobacco products	55 (22.5)
Tobacco with other grocery items	189 (77.5)

The majority of the study population (n=174; 71.3%) were ≥35 yrs, male participants (n=203; 83.2%), graduate and above (n=59; 24.2%), duration of running the shop ≥10 yrs (n=174; 71.3%), having a permanent or fixed shop (n=211; 86.5%), and selling tobacco with other mixed items (n=189; 77.5%).

Table 2 shows the level of awareness of tobacco vendors about different sections of COTPA related with POS.

**Table 2:** Knowledge of the tobacco vendors about COTPA (n=244) Data has been represented by n and % where n is the number of vendors.

Variable		N (%)
Awareness about tobacco-related legislation		
Yes	192 (78.7)	
No/don’t know	52 (21.3)	
Awareness about not smoking in public places		
Correct	229 (93.9)	
Incorrect	15 (6.1)	
Awareness about fine or penalty for public smoking		
Correct	88 (36.1)	
Incorrect	156 (63.9)	
Awareness about prohibition of sale of tobacco products near to an educational institute		
Correct	150 (61.5)	
Incorrect	94 (38.5)	
Awareness about the minimum distance of selling tobacco products from educational institute		
Correct	7 (2.9)	
Incorrect	237 (97.1)	
Awareness about selling of tobacco products to minor		
Correct	152 (62.3)	
Incorrect	92 (37.7)	
Awareness about promotional advertisement of tobacco products at point of sale (POS)		
Correct	99 (40.6)	
Incorrect	145 (59.4)	
Awareness about maximum number of promotional advertisements at the POS		
Correct	22 (9.0)	
Incorrect	222 (91.0)	
Awareness about maximum size of the promotional board at the POS		
Correct	22 (9.0)	
Incorrect or don’t know	222 (91.0)	
Awareness about the proportion of health warning at tobacco promotional advertisement board at the POS		
Correct	38 (15.6)	
Incorrect or don’t know	206 (84.4)	
Awareness about the signage telling “selling of tobacco to minor is illegal” at the POS		
Correct	139 (57.0)	
Incorrect	105 (43.0)	
Awareness about selling of single cigarette/bidi to customer		
Correct	0 (0.0)	
Incorrect	244 (100.0)	

Majority of the vendors (n=229; 93.9%) were aware that smoking was prohibited at public places followed by awareness about the COTPA (n=192; 78.7%), and about a signage explain that “selling of tobacco to minor is illegal” at POS (n=139; 57.0%). However, correct knowledge about selling of a single bidi or cigarette was nil followed by the minimum distance of selling tobacco products from educational institutes (n=7; 2.9%), followed by awareness about maximum number of promotional advertisement at POS (n=22; 9.0%) and maximum size of the promotional board at POS (n=22; 9.0%).

Table 3 shows that tobacco advertisement material was present in majority of the shops (n=254; 83.9%), but more than maximum permitted numbers (>2) were present in some (n=35; 14.3%), and size was not compliant in some shops (n=85; 34.8%).

**Table 3:** Compliance of the tobacco vendors at POS with COTPA (sections five and six) Data has been represented by n and % where n is the number of vendors; POS: point of sale; COTPA: Cigarettes and Other Tobacco Products Act.

Variable	N (%)	
COTPA (Section 5)		
Tobacco advertisement material at the shop		
Yes	245 (83.9)	
No	47 (16.1)	
Tobacco-related promotional advertisement material (2 or fewer)		
Compliant	209 (85.7)	
Non-compliant	35 (14.3)	
Size of the promotional advertisement		
Small (<60 cm X 90 cm)	159 (65.2)	
Large (≥60 cm X90 cm)	85 (34.8)	
Open display of tobacco products		
Yes	229 (93.9)	
No	15 (6.1)	
Display board showing all the pictorial depiction of the ill effects of tobacco on health		
Compliant	13 (5.3)	
Non-compliant	231 (94.7)	
Display of signage showing “selling of tobacco to minor is illegal” at POS		
Compliant	4 (1.6)	
Non-compliant	240 (98.4)	
COTPA (Section 6)		
Tobacco products are sold by minor		
Yes		19 (7.8)
No		225 (92.2)
Tobacco products are bought by minor		
Yes	8 (3.3)	
No	236 (96.7)	
Location of the vendor is within 100 yards of radial distance from the educational institute’s main gate or boundary		
Yes	47 (19.3)	
No	197 (80.7)	

Open display of tobacco products was present in most shops (n=229; 93.9%) whereas signage showing the pictorial depiction of the ill-effects of tobacco on health (n=13; 5.3%) and “selling of tobacco to minor is illegal” (n=4; 1.6%) was present only in a few shops. Tobacco products being sold to (n=19; 7.8%) and bought by (n=8; 3.3%) minors was observed in some cases, whereas a school was within 100 yards of the shop in some cases (n=47; 19.3%).

Table 4 shows that the maximum and minimum knowledge scores attained by the vendors was from one to 10 with a mean of 4.5± 1.82.

**Table 4:** Categorization of knowledge and compliance to the COTPA (sections five and six) among vendors (n=244) Data has been represented by n and % where n is the number of vendors; POS: Point of sale; COTPA: Cigarettes and Other Tobacco Products Act.

Variable	N (%)
Knowledge score of vendors	
Adequate (7-12)	47 (19.3)
Inadequate (0-6)	197 (80.7)
Compliance at POS	
Adequate (5-9)	124 (50.8)
Inadequate (0-4)	120 (49.2)

Overall, several vendors (n=197; 80.7%) had an inadequate knowledge score whereas nearly half of them had an inadequate compliance score (n=120; 49.2%).

In the univariate and multivariable logistic regression analyses, we examined the association between the tobacco vendors' knowledge of the COTPA and their sociodemographic characteristics and the operational profile of their shops. There was not much multicollinearity among the independent variable because lowest tolerance value is 0.637 and range of variance inflation factor (VIF) is 1.132-1.187. Unadjusted odds ratio (UOR) showed that gender (3.505 (1.033-11.896)), education level of the vendors (4.975 (2.018-12.264)) and type of seller (2.091 (1.041-4.201)) had significant association with adequate knowledge scores, whereas after adjustment (AOR), education (4.117 (1.377-12.310), p=0.011) and type of seller (2.606 (1.209-5.615), p=0.014) had significant association with vendor knowledge score regarding the COTPA. As the level of education increased (AOR=4.11), there was a significant (p=0.011) increase in the level of knowledge score among the vendors. Similarly vendors exclusively selling tobacco products (AOR=2.60) had significantly (p=0.014) higher knowledge score compared to those who were selling mixed products. Age, gender, and duration of running the shop did not hold any significant association with adequate knowledge score on COTPA (Table 5).

**Table 5:** Univariate and multivariable logistic regression for association between the knowledge of tobacco vendors about COTPA and sociodemographic factors as well as operational profile of the shops Data has been represented by n and % where n is the number of vendors. In the univariate logistic regression, UOR is unadjusted odds ratio. In the multivariate logistic regression, AOR is adjusted odds ratio. In the multivariable logistic regression, the dependent variable is the knowledge score where adequate knowledge score code=1, and inadequate knowledge score code=0. A p-value ≤0.05 was considered statistically significant. 95% confidence interval was considered along with odds ratio. The model was well fitted with a non-significant value (P=0.702) using Hosmer and Lemeshow test. After controlling for the predictors, the model explained between 29.5% (Cox and Snell R2) and 35.4% (Nagelkerke R2) of the variance of the knowledge score in the study participants and correctly classified 65.0% of the cases.

Variable	Category	Adequate knowledge score n (%)	Inadequate knowledge score n (%)	Unadjusted odds ratio (UOR) (95% CI)	Beta coefficient	Adjusted odds ratio (AOR) (95% CI)	p-value
Age (in years)	≥35	31 (17.8)	143 (82.2)	Reference		Reference	0.549
	<35	16 (22.9)	54 (77.1)	0.732 (0.371-1.444)	-0.345	0.708 (0.273-1.840)	
Gender	Male	44 (21.7)	159 (78.3)	Reference		Reference	0.549
	Female	3 (7.3)	38 (92.7)	3.505 (1.033- 11.896)	-0.455	0.635 (0.144-2.804)	
Education	Up to primary school	6 (6.7)	83 (93.3)	Reference		Reference	0.011
	Above high school	41 (26.5)	114 (73.5)	4.975 (2.018-12.264)	1.415	4.117 (1.377-12.310)	
Duration of running the shop	≥10yrs	28 (16.1)	146 (83.9)	Reference		Reference	0.081
	<10 yrs	19 (27.1)	51 (72.9)	0.515 (0.265-1.000)	0.835	2.305 (0.903-5.886)	
Type of shop	Movable	6 (18.2)	27 (81.8)	Reference		Reference	0.950
	Permanent or fixed	41 (19.4)	170 (80.6)	1.085 (0.421-2.801)	-0.033	0.968 (0.348-2.692)	
Type of seller	Tobacco with other grocery items	31 (16.4)	158 (83.6)	Reference		Reference	0.014
	Only tobacco products	16 (29.1)	39 (70.9)	2.091 (1.041-4.201)	0.958	2.606 (1.209-5.615)	

In the univariate and multivariable logistic regression analyses, we investigated the association between tobacco vendors' compliance with COTPA (sections five and six) at POS and their sociodemographic characteristics and the operational profile of their shops. Multicollinearity was checked among independent variables and lowest tolerance value was 0.528 and range of variance inflation factor (VIF) is 1.115-2.123. UOR and AOR showed that the type of shop (2.343 (1.055-5.202)) and the type of seller (2.093 (1.088-4.026)) had a significant association with an adequate compliance score. Those who had a permanent or fixed tobacco shop (AOR=2.09) has significantly (p=0.036) more compliance to sections five and six of COTPA. Similarly, those who were selling mixed products (tobacco along with other grocery items) (AOR=1.75) had a significantly (p=0.027) higher compliance score compared to those selling tobacco products alone. Age, gender, duration of running the shop, and knowledge score of the vendors does not hold any significant association with an adequate compliance score on COTPA (sections five and six; Table 6).

**Table 6:** Univariate and multivariable logistic regression for the association between the compliance of tobacco vendors at POS with COTPA (sections five and six) and the sociodemographic factors as well as the operational profile of the shops Data has been represented by n and % where n is the number of vendors. In the univariate logistic regression, UOR is unadjusted odds ratio, and in the multivariable logistic regression, AOR is the adjusted odds ratio. In multivariable logistic regression, the compliance score is the dependent variable, where an adequate compliance score code=1, and an inadequate compliance score code=0. A p-value ≤0.05 was considered statistically significant. 95% confidence interval was considered along with odds ratio. The model was well fitted with a non-significant value (P=0.501) using Hosmer and Lemeshow test. After controlling for the predictors, the model explained between 27.5% (Cox and Snell R2) and 31.2% (Nagelkerke R2) of the variance of knowledge score in the study participants and correctly classified 59.0% of the cases.

Variable	Category	Adequate compliance n (%)	Inadequate compliance n (%)	Unadjusted odds ratio (95% CI)	B coefficient	Adjusted odds ratio (95% CI)	p-value
Age (in years)	<35	32 (45.7)	38 (54.3)	Reference		Reference	0.646
	>35	92 (52.9)	82 (47.1)	1.332 (0.764-2.324)	0.181	1.19 (0.553-2.597)	
Gender	Female	23 (56.1)	18 (43.9)	Reference		Reference	0.458
	Male	101 (49.8)	102 (50.2)	0.775 (0.394-1.523)	-0.316	0.729 (0.316-1.680)	
Education	Up to high school	48 (53.9)	41 (46.1)	Reference		Reference	0.579
	Above high school	76 (49.0)	79 (51.0)	0.822 (0.487-1.385)	-0.187	0.830 (0.429-1.605)	
Duration of running the shop	<10 yrs.	33 (47.1)	37 (52.9)	Reference		Reference	0.600
	≥10yrs	91 (52.3)	83 (47.7)	1.229 (0.705 -2.143)	0.208	1.231 (0.567-2.673)	
Type of shop	Movable	11 (33.3)	22 (66.7)	Reference		Reference	0.036
	Permanent or fixed	113 (53.6)	98 (46.4)	2.306 (1.065-4.994)	0.851	2.343 (1.055-5.202)	
Type of seller	Only tobacco product	22 (40.0)	33 (60.0)	Reference		Reference	0.027
	Tobacco with other grocery items	102 (54.0)	87 (46.0)	1.759 (0.955-3.239)	0.738	2.093 (1.088-4.026)	
Knowledge score	Inadequate knowledge	97 (49.2)	100 (50.8)	Reference		Reference	0.098
	Adequate knowledge	27 (57.4)	20 (42.6)	1.392 (0.732-2.645)	0.585	1.794 (0.898-3.586)	

## Discussion

The study conducted in Raebareli, Uttar Pradesh, provides critical insights into the knowledge and compliance levels of tobacco vendors with sections five and six of COTPA 2003, a cornerstone of India’s tobacco control framework. The findings reveal significant gaps in both awareness and adherence to these regulations at the POS, underscoring the challenges of implementing tobacco control policies in tier II cities like Raebareli.

Knowledge of tobacco vendors

The study found that the majority of tobacco vendors in Raebareli had inadequate knowledge of COTPA, with a mean knowledge score reflecting limited understanding of specific provisions. Notably, while more than 90% were aware of the prohibition on smoking in public places, awareness of critical POS-specific regulations, such as the minimum distance for selling tobacco near educational institutions. The ban on selling single cigarettes or bidis was alarmingly low. These findings align with other studies in India, such as a mixed-method study in Puducherry, which reported that only 45.7% of vendors were aware of the prohibition on tobacco sales within 100 yards of educational institutions, and a mere 2.6% adhered to the ban on loose cigarette sales [12]. Similarly, a study in Moradabad, Uttar Pradesh, noted poor vendor knowledge of COTPA specifics, despite general awareness of anti-tobacco laws [13]. However, a recent study conducted in four districts of south Indian states shows a contrasting finding, probably due to a difference in overall education level as well as the enforcement effort of the local government system [14].

The low knowledge levels in Raebareli may stem from the limited outreach and education efforts targeting vendors, particularly in smaller cities where enforcement mechanisms are often weaker than in metropolitan areas. The multivariable logistic regression analysis revealed that higher education levels (AOR=4.117, p=0.011) and exclusive tobacco vending (AOR=2.606, p=0.014) were significantly associated with better knowledge scores. This suggests that vendors with more education may have greater access to or comprehension of regulatory information, while those solely selling tobacco may be more exposed to enforcement activities or industry-related communications. These findings contrast with a study in Khammam, Andhra Pradesh, where older vendors were less knowledgeable, possibly due to generational differences in media exposure [11]. The gender disparity, where male vendors showed higher awareness (UOR=3.505), though not significant after adjustment, may reflect the predominance of male vendors and their greater interaction with customers and authorities.

Compliance with COTPA sections

Compliance with COTPA sections five and six at POS in Raebareli was suboptimal, and nearly half of vendors exhibited inadequate compliance. Specific violations included the presence of tobacco advertisement materials in 83.9% of shops, with 14.3% exceeding the permitted number of advertisements (>2) and 34.8% using oversized boards. Open display of tobacco products was nearly ubiquitous (93.9%), and compliance with health warning signage (5.3%) and notices prohibiting sales to minors (1.6%) was minimal. Additionally, 19.3% of shops were located within 100 yards of educational institutions, and sales to (3.3%) and by (7.8%) minors were observed, albeit at lower rates than other violations.

These results are consistent with broader trends in India. A cross-sectional study across five states, including Uttar Pradesh, reported that 96% of educational institutions in Prayagraj violated section six b [15]. Similarly, a study in Delhi found only 52.85% compliance with section six b and 68.57% with section six a, highlighting persistent challenges in enforcing restrictions on sales to minors and near educational institutions [16]. The high prevalence of open tobacco displays in Raebareli mirrors findings from Karnataka study where 94% of vendors sold loose tobacco [17]. The low compliance with health warning signage and notices about sales to minors further corroborates evidence from Mumbai, where only 10% of vendors displayed required signage [4].

The logistic regression analysis indicated that permanent shops (AOR=2.343, p=0.036) and vendors selling tobacco alongside other grocery items (AOR=2.093, p=0.027) were more likely to comply with COTPA provisions. This may reflect greater visibility and accountability for fixed shops, which are easier to monitor, and the diversified income sources of mixed-item vendors, reducing reliance on tobacco sales. However, the lack of association between knowledge and compliance (AOR=1.794, p=0.098) suggests that awareness alone does not translate into adherence, pointing to enforcement gaps or external pressures, such as economic incentives from the tobacco industry.

Global evidence suggests that countries with formal guidelines and specified timelines for FCTC Article 13 implementation have made more rapid progress. Many low- and middle-income countries (LMICs), including India, struggle with enforcement. Present study findings mirror challenges observed in many South Asian countries. For instance, a study in Lebanon reported low compliance with POS advertising restrictions [18]. In Europe and New Zealand, high compliance with POS restrictions has been linked to lower tobacco use, particularly among youth [19]. In high-income countries (HICs) like the United Kingdom, Australia, and Canada, Article 16 is implemented through stringent age-of-sale laws, typically setting the minimum age at 19, coupled with robust enforcement mechanisms [20], whereas Uganda and the Republic of Moldova have also introduced comprehensive tobacco legislation, including Article 16 provisions, but face similar enforcement issues [21,22].

Limitations

The study’s use of convenience sampling and absence of sampling frame (unavailability of tobacco vendors licensing), high refusal rate for participation may limit generalizability, as it may not fully represent all vendors in Raebareli. The exclusion of large shops and malls also restricts the scope of findings. Additionally, qualitative studies exploring vendors’ perceptions of enforcement barriers, could provide deeper insights into non-compliance drivers. Longitudinal studies assessing the impact of targeted interventions on compliance and tobacco use rates would further strengthen the evidence base.

## Conclusions

This study in Raebareli underscores significant deficiencies in tobacco vendors’ knowledge and compliance with COTPA sections five and six, reflecting broader systemic challenges in India’s tobacco control landscape. While education and shop type influence knowledge and compliance, systemic enforcement gaps persist. It also highlights that India’s legal framework is robust, but enforcement and awareness among vendors are critical barriers. Strengthening awareness, enforcement, tobacco vendor licensing, and stakeholder collaboration is critical to reducing tobacco accessibility, particularly among youth, in such tier II cities. To address these gaps, a multifaceted approach is necessary. First, targeted education campaigns for vendors, emphasizing COTPA’s specific provisions, could improve knowledge. These campaigns should leverage local languages and media, as younger vendors in Raebareli were more exposed to information via television and social media. Second, stricter enforcement mechanisms, including regular compliance checks and penalties, are essential.

## Appendices

**Table 7:** Questionnaire/proforma used in the study

Demographic characteristics of the participants and Tobacco vendors shops profile		
Sl Number:	Name: (optional)………………………….	Gender:
Age (yrs.)…………………………….	Education: …………………………….	Duration of running the shop: …………
Type of point of sale: Temporary/ permanent	Type of seller: Only tobacco products/ Tobacco with other grocery items	

Sl. No	Knowledge of the tobacco vendors about COTPA Act						
	Statement	Yes		No	Don’t Know	Score	Maximum Attainable score
1	Have you heard of any law/ act related to tobacco consumption?	1		0	0	Yes=1, No=0	1
2	Can you smoke/ consume tobacco in public places in India?	0		1	0	Yes=0, No=1	1
3	Is there any fine for public smoking in India?	1		0	0	Yes=1, No=0	1
4	Can you sell tobacco products near the educational institute?	0		1	0	Yes=0, No=1	1
5	What is the minimum distance for selling tobacco products from the school?	100 yards or more=1		Incorrect=0	0	Yes=1, No=0	1
6	Can you sell tobacco products to minors or <18 yrs?	0		1	0	Yes=0, No=1	1
7	Can you use advertisements for the promotion of tobacco at point of sale?	1		0	0	Yes=1, No=0	1
8	What should be the maximum number of promotional advertisements at POS of tobacco product	2 or less=1		0	0	Yes=1, No=0	1
9	What should be the maximum size of promotional advertisement at POS of tobacco product	60X90 cm= 1		0	0	Yes=1, No=0	1
10	How much proportion of tobacco advertisement board should contain health warning at POS? (Correct=1, Incorrect=0)	25% of the display area =1		0	0	Yes=1, No=0	1
11	Does it necessary to display a signage telling ‘selling tobacco to minors is illegal”?	1		0	0	Yes=1, No=0	1
12	Can you sell a single Bidi/cigarette to a customer?	0		1	0	Yes=0, No=1	1
Maximum score							12
Practice/compliance related to COTPA ACT (Section 5 & 6)							
			Present/Yes		Absent/No	Score	Maximum Attainable score
1	Tobacco advertisement material in the shop		0		1	Absent=1, Present=0	1
2	Tobacco related promotional advertisement material 2 or less		1		0	Yes=1, No=0	1
3	Size of the promotional advertisement		Small (<60X90 cm) =1		Large (>60X90 cm) =0	Small=1, Large =0	1
4	Open display of tobacco products		0		1	Yes=0, No=1	1
5	Display of board showing all pictorial depictions of the ill effects of tobacco use on health		1		0	Yes=1, No=0	1
6	Display of signage telling ‘selling tobacco to minor is illegal'		1		0	Yes=1, No=0	1
7	Presence of tobacco selling by minors (<18 yrs)		0		1	Yes=0, No=1	1
8	Tobacco products are bought by minor (<18 yrs)		0		1	Yes=0, No=1	1
9	Location of the vendor is within 100 yards of radial distance from the educational institute’s main gate or boundary.		0		1	Yes=0, No=1	1
Maximum score							9
